# Supplementary figures and images for: Emergent spatial structure in the gut microbiota is driven by bacterial growth and gut contractions
Source: PLoS Biol. 2026 Apr 24;24(4):e3003772. doi: 10.1371/journal.pbio.3003772 (PMC13128125; doi:10.1371/journal.pbio.3003772)

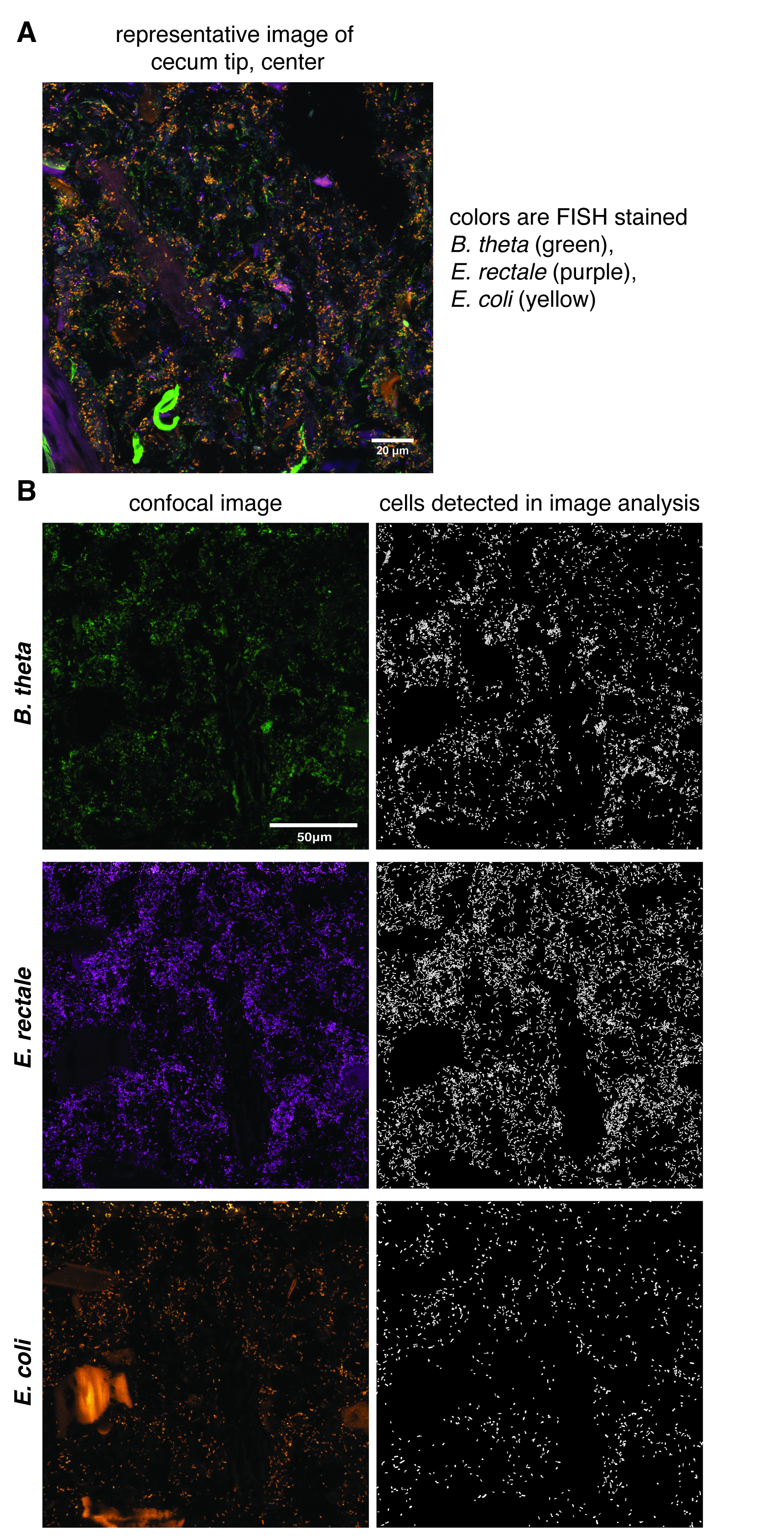

Supplement: S1 Fig — (A) Representative image of the cecum tip, center, of a 3MM mouse. (B) Left column shows confocal microscopy images for the three bacterial species in the 3MM microbiota, detected by FISH probes in fixed cecum content. Right column shows the binarized images resulting from image analysis. The data underlying this Figure can be found in the data repository accompanying this paper under the DOI: https://doi.org/10.5281/zenodo.19422141. (TIF) [file pbio.3003772.s001.tif]

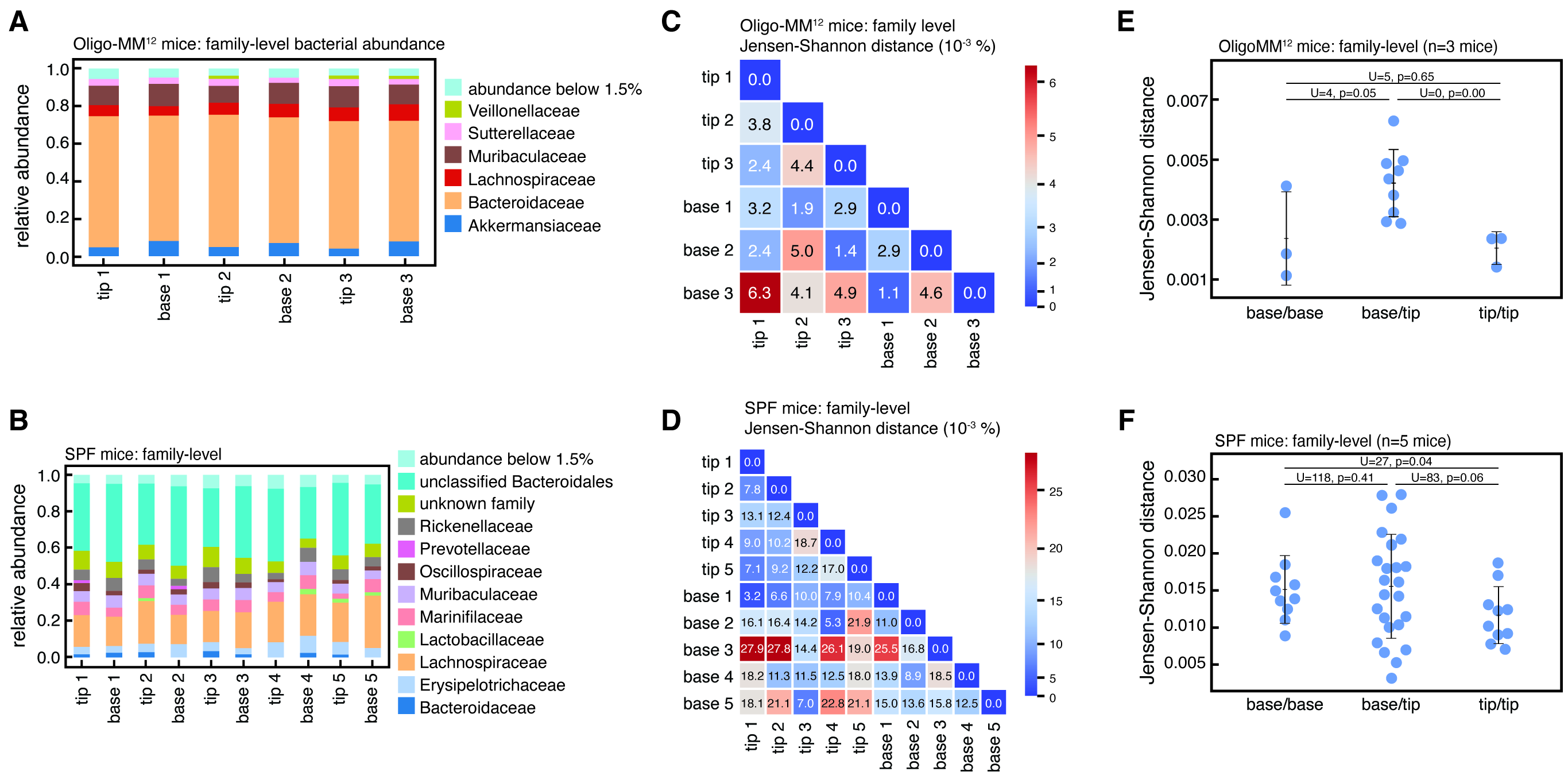

Supplement: S2 Fig — (A) Results of 16S sequencing of cecum content of OligoMM12 (n = 3) and (B) SPF mice (n = 5) at the family-level. Tip and base samples of the same cecum were sequenced separately. (C) Jensen-Shannon distances between each sequenced tip and base for OligoMM12 and (D) SPF mice at the family-level. (E) Plots of base/base, base/tip, and tip/tip Jensen-Shannon distances across all mice in the OligoMM12 group and (F) the SPF group. Significance was tested with a one-sided Mann–Whitney U test. The data underlying this Figure can be found in the data repository accompanying this paper under the DOI: https://doi.org/10.5281/zenodo.19422141. (TIF) [file pbio.3003772.s002.tif]

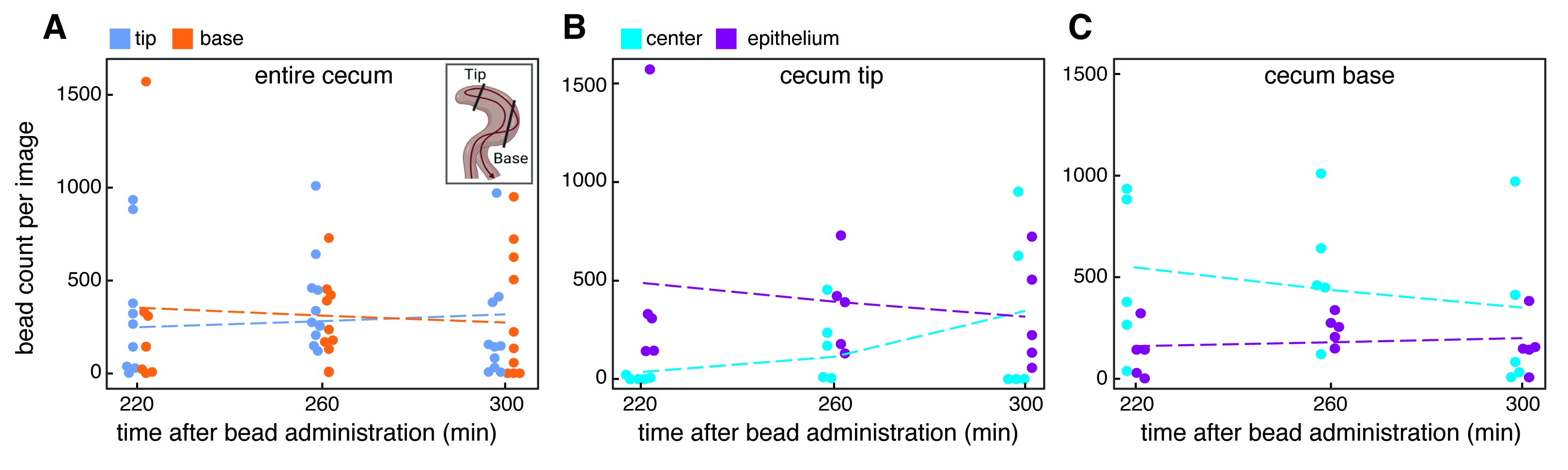

Supplement: S3 Fig — (A) Bead count in cecum tip (blue) an cecum base (orange) for beads administered 220 min (green beads), 260 min (blue beads), and 300 min (red beads) before sacrifice. Insert shows a schematic representation of the cecum, highlighting location of tip and base. (B) Bead count in the cecum tip, in images taken close to the epithelium (purple) or in the center (blue) of the cecum, depending on the time after bead administration. (C) Bead count in the cecum base, in images taken close to the epithelium (purple) or in the center (blue) of the cecum, depending on the time after bead administration. The insert in (A) was created using BioRender (https://biorender.com/58n1m7d). The data underlying this Figure can be found in the data repository accompanying this paper under the DOI: https://doi.org/10.5281/zenodo.19422141. (TIF) [file pbio.3003772.s003.tif]

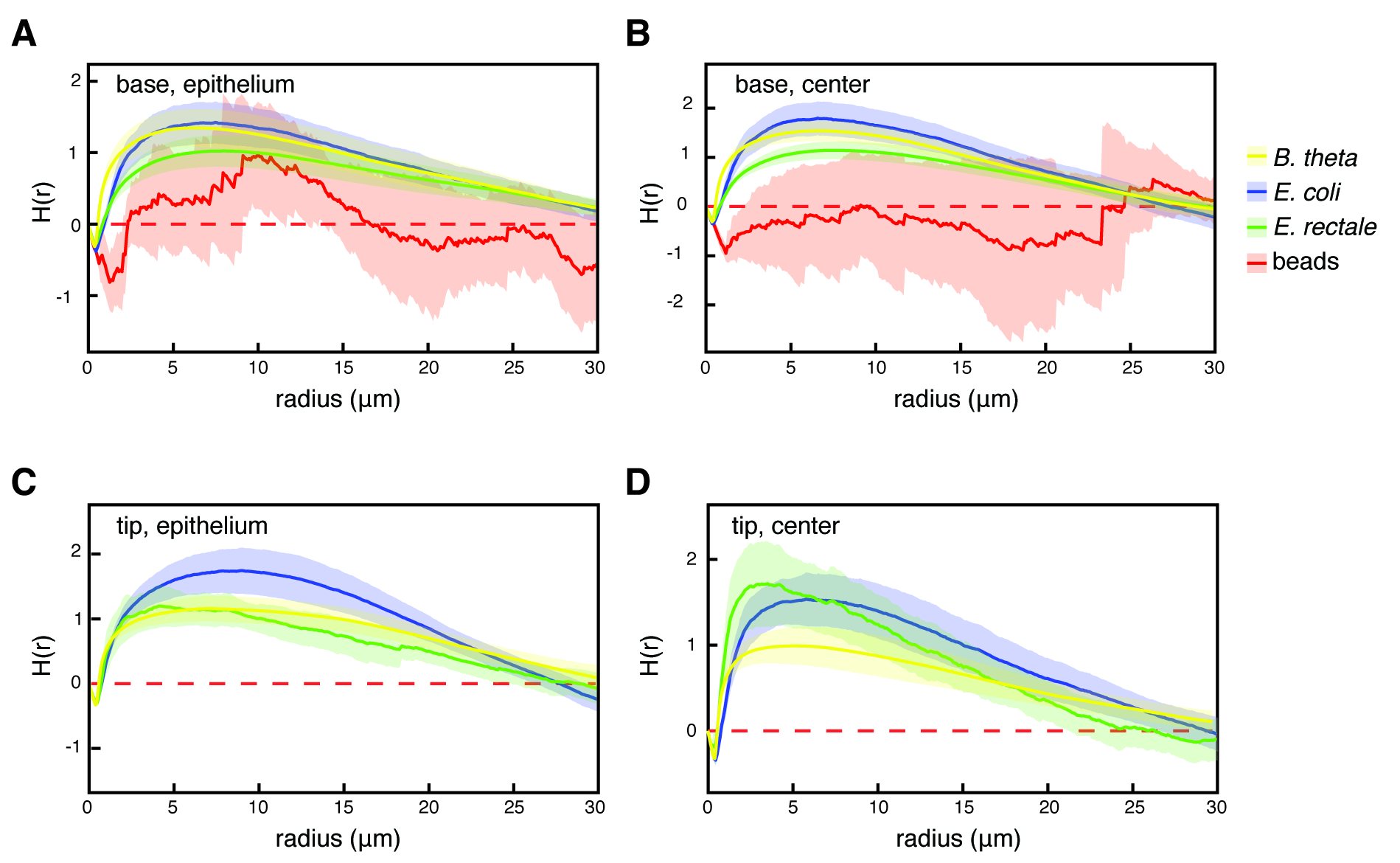

Supplement: S4 Fig — Results for all three 3MM species when cell distribution was analyzed using the inhomogeneous H function. Shaded regions indicate 95% CI (n = 6). Red dashed lines indicate the theoretical value for complete spatial randomness. Locations are (A) base, epithelium, (B) base, center, (C) tip, epithelium, and (D) tip, center. H(r) functions for all bacterial species are significantly different from complete spatial randomness (p < 0.01). With the exception of E. rectale at the epithelium of the cecum base, H(r) functions for all bacteria data were significantly different from H(r) function for beads (p < 0.01). H(r) functions for beads are significantly different from complete spatial randomness at the base epithelium (p = 0.04), but not at the base center. No beads were found at the cecum tip. Bonferroni-corrected Studentized permutation tests were used for statistical comparisons between H(r) functions. The data underlying this Figure can be found in the data repository accompanying this paper under the DOI: https://doi.org/10.5281/zenodo.19422141. (TIF) [file pbio.3003772.s004.tif]

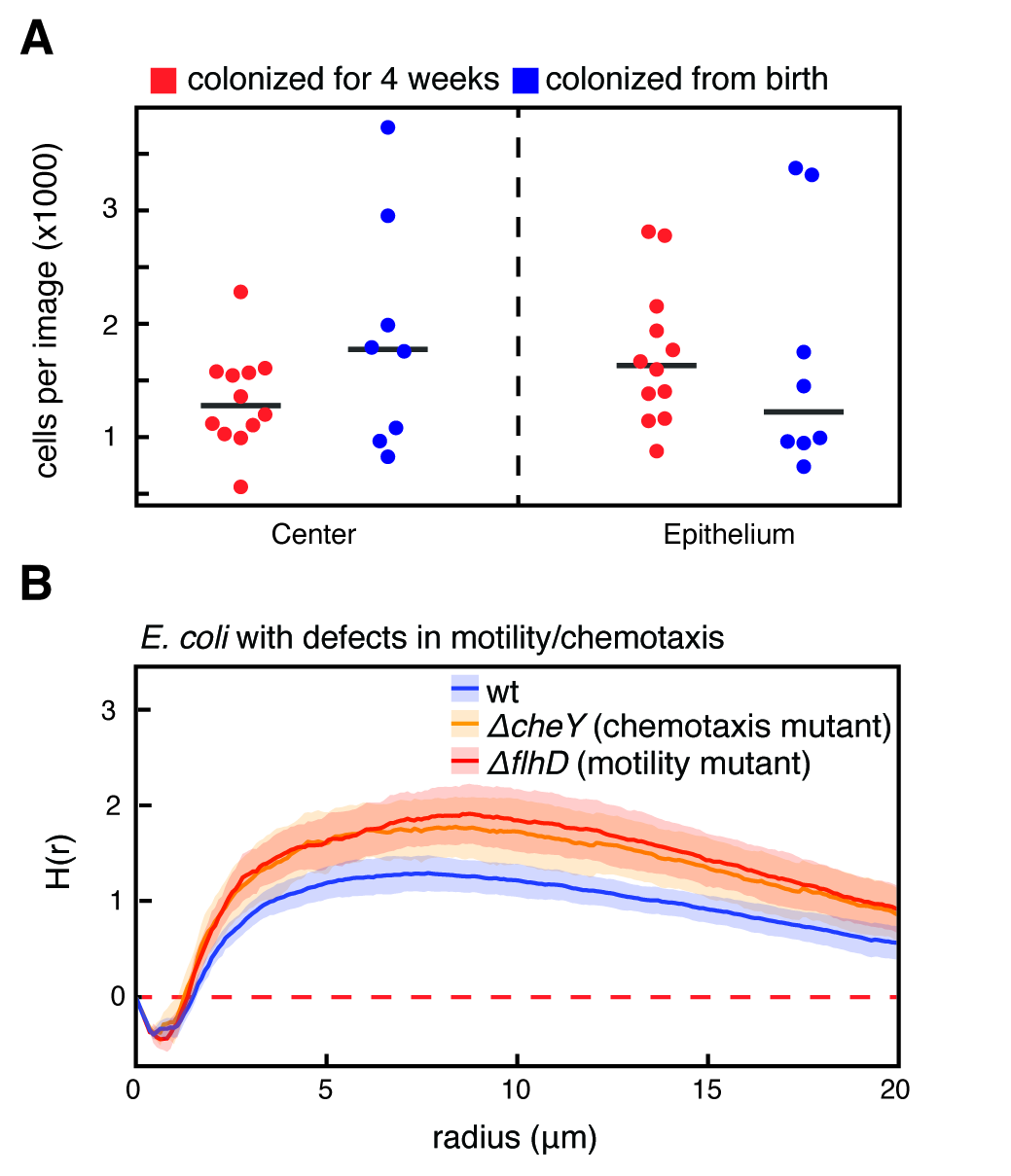

Supplement: S5 Fig — (A) Cell counts, based on image analysis, for E. coli in mice that were colonized from birth with the 3MM microbiota (blue), and mice that were colonized for 4 weeks (red). Only mice that were colonized for 4 weeks have sIgA antibodies against E. coli. (B) Results for spatial distribution of wt (blue), ΔcheY mutant (yellow), and ΔflhD mutant E. coli cells in the cecum ex-germ-free mice, using the inhomogeneous H function (n = 12, 4 in each group, cecum base epithelium data shown). All groups show significant clustering (p < 0.01), and there is no significant difference in clustering between wild type and ΔcheY (p > 0.05), but between wild type and ΔflhD (p = 0.12). The form of the H(r) function suggests that ΔflhD clusters more than wild type, indicating that flagella might be important for cluster dispersal rather than cluster formation. Bonferroni-corrected Studentized permutation tests were used for statistical comparison between H(r) functions. The data underlying this Figure can be found in the data repository accompanying this paper under the DOI: https://doi.org/10.5281/zenodo.19422141. (TIF) [file pbio.3003772.s005.tif]

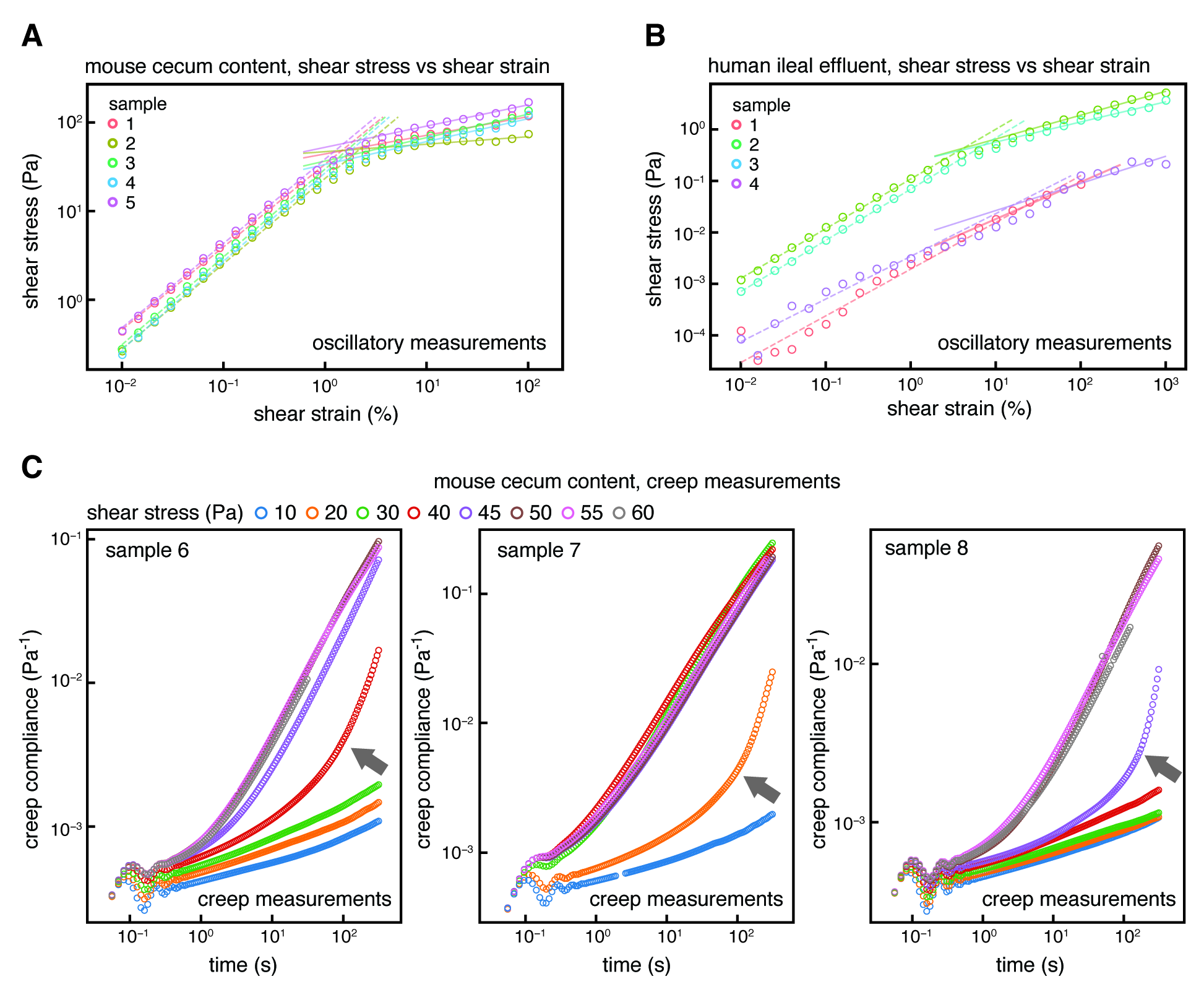

Supplement: S6 Fig — (A) Shear stress as a function of shear strain in five samples of 3MM mouse cecum content in oscillatory measurements (same dataset as shown in Fig 4A). The lines are power-law fits of the behavior well above (full lines) and below (dashed lines) the yield point, and the Y coordinate of their intersection points corresponds to the yield limit (yield stress values: 47.79 Pa, 50.13 Pa, 39.66 Pa, 36.25 Pa, 58.21 Pa, for samples 1–5, respectively). (B) Same analysis as (A), but for four samples of human ileal effluent (same dataset as shown in Fig 4E). Yield stress values are 0.03 Pa, 0.44 Pa, 0.50 Pa, 0.03 Pa, for samples 1–4, respectively. (C) Creep compliance as a function of time for three independent samples of 3MM mouse cecum content. The arrows indicate the shear stress where creep compliance increases linearly with time (indicating fluidization of the material), the yield limit. The data underlying this Figure can be found in the data repository accompanying this paper under the DOI: https://doi.org/10.5281/zenodo.19422141. (TIF) [file pbio.3003772.s006.tif]

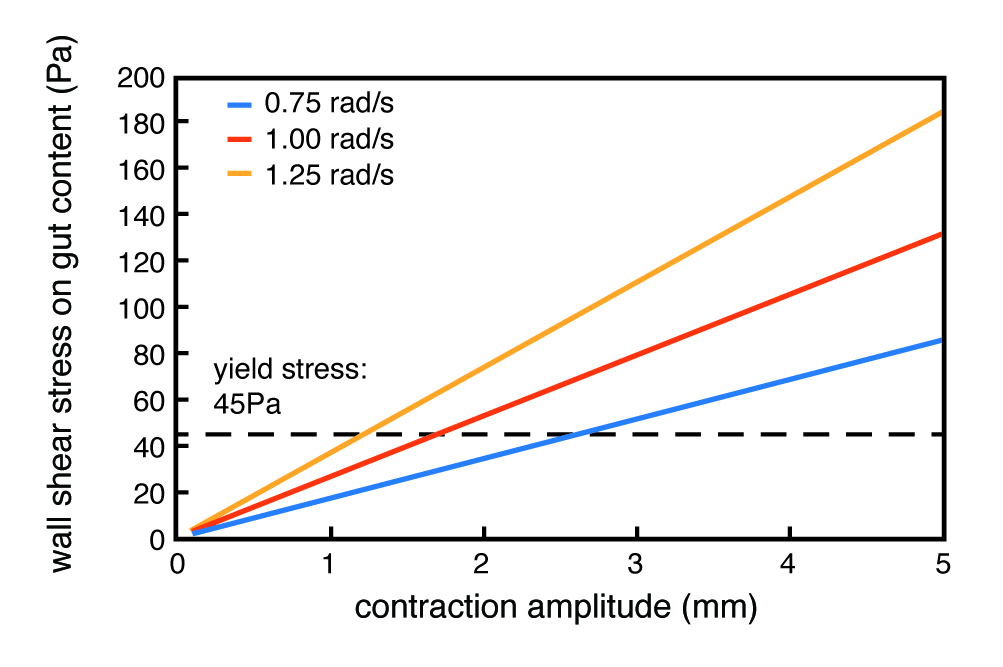

Supplement: S7 Fig — The shown results are based on Stokes’ second problem and take measured values for viscosity and density of gut content into account. Dashed line indicates measured yield stress of gut content (45 Pa). The data underlying this Figure can be found in the data repository accompanying this paper under the DOI: https://doi.org/10.5281/zenodo.19422141. (TIF) [file pbio.3003772.s007.tif]

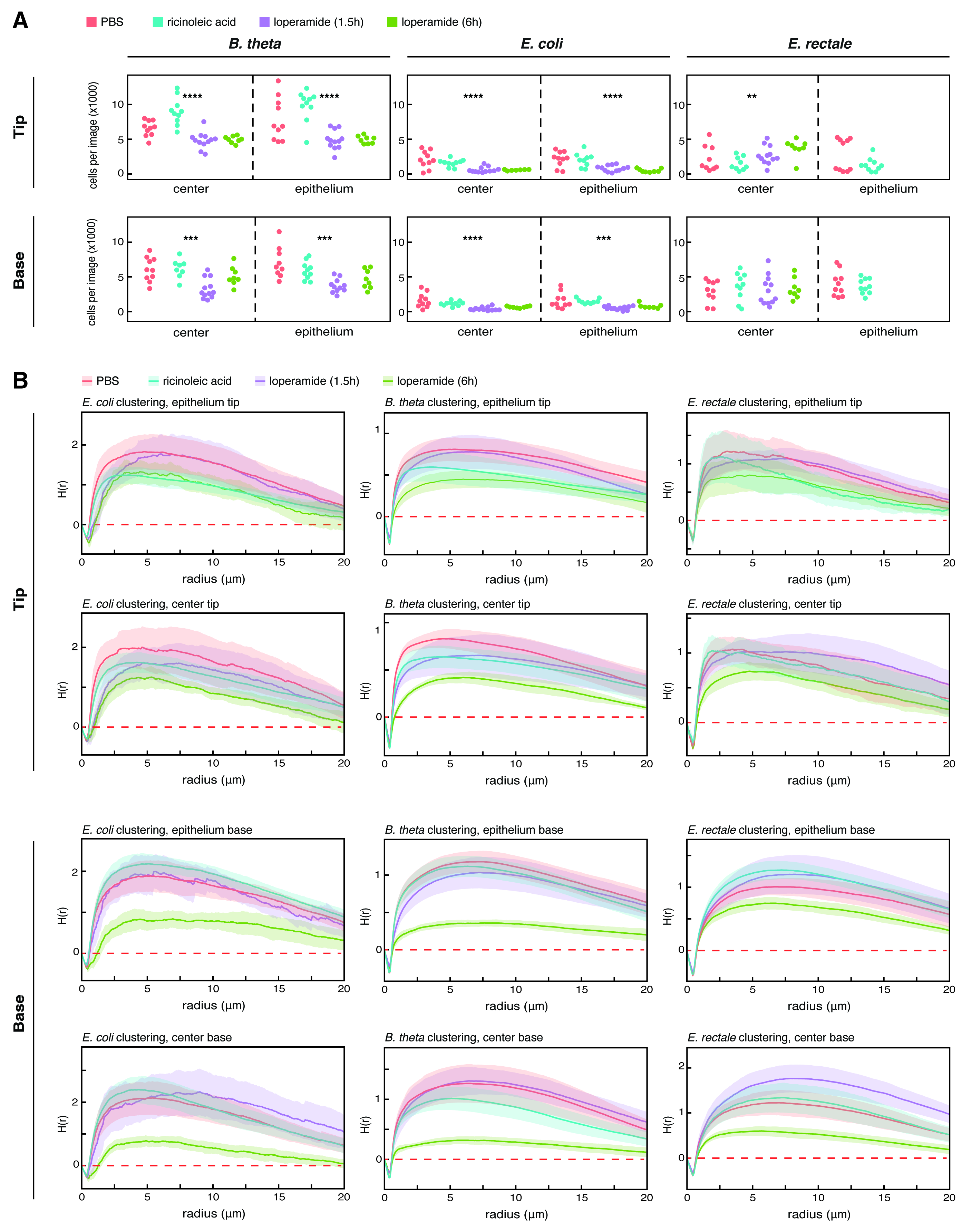

Supplement: S8 Fig — (A) Cell counts on microscopy images in ricinoleic acid and PBS treated groups. In most cases (all except E. rectale base center) where we have data for all 4 treatment groups, significant differences exist in cell counts between groups (ANOVA, **, ***, **** correspond to p < 0.01, 0.001, and 0.0001, respectively) (B) Clustering analysis using the inhomogeneous H function for all three 3MM strains after IP injection of PBS (red), ricinoleic acid (cyan), and loperamide (purple) 1.5 h after treatment, and for loperamide 6 h after treatment (green), for different locations in the cecum. Studentized permutation tests were used to assess whether curves were significantly different, and p values were Bonferroni-corrected for the number of comparisons (3 in each case: control versus ricinoleic acid, control versus loperamide 1.5 h, control versus loperamide 6 h). For E. coli, the following curves were significantly different: control versus loperamide 6 h (base center; p = 0.003), control versus loperamide 6 h (base epithelium; p = 0.006), control versus loperamide 6 h (tip center; p = 0.006). For B. theta, we found significant differences between the following curves: control versus loperamide 6 h (base center; p = 0.003), control versus loperamide 6 h (base epithelium; p = 0.003), control versus loperamide 6 h (tip center, p = 0.003), control versus loperamide 6 h (tip epithelium, p = 0.024). For E. rectale, the following comparisons differed significantly: control versus loperamide 6 h (base center; p = 0.003), control versus loperamide 6 h (base epithelium; p = 0.003), control versus loperamide 6 h (tip epithelium, p = 0.039). Of note, the comparisons between control and ricinoleic acid that were significantly different with p < 0.05 without Bonferroni correction (E. coli: tip epithelium, p = 0.02; B. theta: tip epithelium, p = 0.023) lost that significance level after multiple testing correction for three tests. The data underlying this Figure can be found in the data [file pbio.3003772.s008.tif]
