# Supplementary material for: Emergent spatial structure in the gut microbiota is driven by bacterial growth and gut contractions
Source: PLoS Biol. 2026 Apr 24;24(4):e3003772. doi: 10.1371/journal.pbio.3003772 (PMC13128125; doi:10.1371/journal.pbio.3003772)
Supplement: Attachment — Submitted filename: response_to_reviewers.pdf [file pbio.3003772.s011.pdf]

Point-by-point responses to the editor's and reviewers' comments

Our responses are in blue italics. We also provide a document that has all changes highlighted.

Summary of our response

We would like to thank the editor and the three reviewers for their careful reading of our work and their constructive and positive remarks. In response to the reviewers' comments, we have extensively edited the text to improve clarity. We have added more image data to the new version and added three experimental datasets. First, we included a second method to determine the yield limit of mouse cecum content (creep rheology) and re-analyzed the oscillatory rheology data from mouse and human gut content to quantify this important property. Second, we have added two datasets on loperamide treated mice (analyzed 1.5h and 6h after treatment). While their outcomes are somewhat surprising, they do nicely illustrate that spatial structure in the gut lumen can indeed be manipulated through mild pharmacological interventions. Please find our detailed responses to all comments below.

Editor

In light of the reviews, which you will find at the end of this email, we would like to invite you to revise the work to thoroughly address the reviewers' reports. While two reviewers are mostly positive, there are several important concerns raised. Reviewer 1 mentions that while the manuscript relies heavily on images, they provide too few of them.

In response to the editor's and the reviewers' comments, we have added more images. Specifically, we have moved microscopy images from the old Figure S4 to Figure 3C, and added the images in FigureS1A, Figure3F, I and Figure4D.

Reviewer 2 says that some of the conclusions are rather speculative, and requires further statistical analysis as well as further image-based analyses. As it is, the reviewer thinks that the role of digesta rheology still remains an open question and requires further experimental support.

We have substantially adapted the language in response to this reviewer's comments, and added more analysis and data on rheology of gut content (Figure S6).

Reviewer 3 thinks the study may not reflect the biology of complex communities, and suggests additional experiments like testing the role of motility in clustering, further testing the model with other alterations like fiber content.

We agree with the reviewer, and discuss these limitations in the new version of the manuscript.

We agree with all reviewer concerns and would require some additional experimental revisions to address them, as we consider that this would strengthen the work.

IMPORTANT: after discussion with the Academic Editor and the reviewers, we would strongly encourage the loperamide experiment suggested by Reviewer 3, which we think can also help in addressing some concerns of Reviewer 2.

We have performed the suggested experiment using two different time courses and animal cohorts (Figure 4C and FigureS8), and discuss it extensively in the text.

Reviewer #1:

In this lovely manuscript, Greter and colleagues leverage a diversity of experimental and computational techniques to explore multiple properties of the spatial organization of the murine gut microbiome and the molecular and mechanical forces that shape this organization. First, the authors use FISH to image the distribution of a model 3-member microbiota comprised of E. coli, B. thetaiotaomicron, and E. ractale. They use these images to precisely quantify the distribution of bacteria along the structure of the murine cecum, with a focus on regions near the center, near the epithelium, and near the cecal tip. They find a non-uniform cecal distribution which they then demonstrate is also present to some degree in both a specific pathogen free (SPF) microbiota as well as the OligoMM12 defined microbiota using sequencing of resected tissue. They then explore the role of peristaltic flow on the distributions by imaging the distribution of fluorescently labeled beads administered at 40 minute intervals within the mice and find that the beads support a order flow into the cecum. They then leverage geospatial statistical methods to characterize the clumpiness of bacteria versus beads and find that the bacteria have a greater degree of clumpiness—i.e. they are more likely to be found to bacteria of the same type than one would predict given the average density—and as an excellent control they show that this is not true for bead distributions. Next they explore a series of potential biological origins for this spatial clumping. They use a intriguing trick to deplete the mice of sIgA—specifically leveraging the fact that mice that were colonized with the three member community from birth do not generate bacterial reactive sIgA whereas germ-free mice that have this community administered do. Remarkably, they find a difference in the spatial distribution in two of the three members between these conditions. Thought they note that this is a relatively smalldifference, and they conclude that sIgA is not responsible for clumping. They also explore the role of motility, but find that in E. coli deficient for flagella production they still see similar distributions. To explore the role of growth, they modify the metabolic niche by exploring the distribution of B. theta in a more diverse microbiota—OligoMM12—with the idea being that with increased competition for nutrients, B. theta likely has a lower growth rate. They still see clustering, but the extent was more greatly reduced, suggesting that growth is indeed responsible for clustering. Finally, they investigate aspects of the cecal environment that could shape cluster size. They make microrheology measurements of cecal content and explore properties under perturbation rates that are likely similar to that seen during normal gut contraction. They then perturb contractions with a small molecule and show that this shifts the degree of clustering in E. coli.

Overall, this paper investigates a fascinating somewhat understudied topic, deploys a variety of methods, and thoroughly examines a series of plausible explanations for the patterns that are seen. I have no major concerns with this work and recommend publication.

We thank the reviewer for their encouraging remarks!

The only minor concern that I would like to share with the authors is described below. I provide this only in the hope that it may help them strengthen an already strong submission.

My minor concern is that for a paper that relies so heavily on images of the spatial distribution of bacteria within the cecum, there are relatively few images provided. The Ripley's K based analysis (the H(r) function) is convincing, yet I think that many readers may share the same curiosity I had regarding the appearance of the actual spatial distributions. Outside of Figure 1B, 2C, S1, and S4B there are no images. Perhaps the authors would consider adding some additional supplemental figures with more example images of the distributions they see, including both replicates of the measurements provided as well as examples from the diversity of measurements analyzed but for which no images were shown.

In response to the reviewer's comments, we have now added example images for more experiments. Specifically, we have moved microscopy images from the old Figure S4 to Figure 3C, and added the images in FigureS1A, Figure3F, I and Figure4D. In addition, we deposit all

raw data that underlies the analyses shown in the manuscript in the publicly available ETH research collection (doi: 10.3929/ethz-b-000665345).

Reviewer #2:

This paper investigates the mechanisms controlling the macro- and microscale organization of the microbiota in the murine caecum. The authors conclude that bacterial growth plays a major role in the formation of bacterial clusters (microcolonies) in the caecum, while gut contractions limit microcolony formation. The study is built upon a series of hypotheses introduced throughout the paper, which support this conclusion. However, the abstract does not clearly reflect the different hypotheses tested and places too much emphasis on the rheological properties of the digesta—an aspect that does not seem essential to explain the observed phenomena (see later). The experiments involve germ-free or gnotobiotic mice colonized with different microbiota. Bacterial localization is assessed on fixed caecal samples, and quantitative observations are performed using confocal microscopy.

We thank the reviewer for their thorough analysis of our manuscript and their constructive remarks. We agree that the abstract of the previous version has somehow overstated the role of rheological properties of cecum content, and we have substantially rewritten the abstract for the new version.

Major comments

Some of the conclusions or interpretations appear somewhat speculative. I detail these points below.

In the Introduction and Discussion sections, the authors discuss how bacterial growth depends on physical heterogeneities in the gut. Given that this study focuses on the caecum, it would be relevant to describe the structural organization of the caecal mucosa (e.g., presence of crypts), as well as what is known about the mucus in this region of the gut (Ref. 47). Can we ignore the role of mucus in the caecum? Are there any oxygen gradients that could influence the microbiota?

We have added a discussion of cecal anatomy, mucus distribution, and the potential for oxygen gradients to form across the epithelium in the cecum (lines 479-486).

A substantial part of the results relies on the interpretation of the Ripley's H function. While it is clear that the sign of H indicates whether bacteria are clustered, randomly distributed, or regularly spaced, can we assign a more quantitative meaning to the value of H? For example, if H at a given r is twice as high in one condition compared to another, does this carry any interpretable significance in terms of spatial organization?

The point about quantitative interpretations of Ripley's H function is well taken. While this is not straight forward, there is some statistical work that tested this specific question, and we have added a discussion, detailing how the radius of Ripley's H function relates to real cluster sizes (lines 518-531). In short, and for the homogeneous version of H(r), the value of H is the radius-normalized number of neighbours found in a circle of radius r around a focal cell. In the inhomogenous H function, this is then weighed by the local density profile of the image. This being said, due to the normalizations involved, a quantitative interpretation of the value of H(r) is complex, and most informative relative to the underlying point density.

Consequently, is it meaningful to perform statistical comparisons on the H(r) profiles?

The studentized permutation test we use for statistical comparisons between H(r) profiles has been developed specifically for this application (Hahn, 2012, Hahn & Jensen 2013) and is

implemented in the spatstat *package in R, which we used for this analysis. We now explain this better in the methods and cite the two papers (lines768-771).*

How did the authors address boundary effects in their spatial analysis?

We used the boundary correction built into the spatstat *R package for H function analysis, specifically the method 'border'; in short, for every given radius r, we only consider points that have a distance >r from the border. However, the different border correction functions available in* spatstat *produced qualitatively similar output when tested, and we used the 'border' method mainly for consistency. We have now clarified this in the methods section (lines 772-776).*

Figure S1 appears to show considerable heterogeneity across the image (possibly due to food particles); could this influence the calculation of the H function?

Indeed, the images show considerable heterogeneity. To address this, we chose to employ the inhomogeneous version of the H function for our analysis, which takes into account local densities on the analyzed images. We have now clarified this in the methods section (lines 744-756).

In general, we thank the reviewer for proposing to include more methodological detail for our use of Ripley's H function. We have now substantially extended the methods section and the discussion to describe the specifics of our analysis and their interpretation.

Although this may not be straightforward, it would be highly informative to provide the probability distribution of aggregate sizes to support comparisons between experimental conditions. Such distributions could potentially be extracted via 2D FFT, image cross-correlation, or other image-based analyses.

We agree that information on aggregate sizes is interesting in this context, and it was not possible to infer that from the data shown in the previous version. While the additional analyses suggested by the reviewer would be interesting, we would argue that with the updated information on the quantitative interpretation of Ripley's H function, aggregate sizes can be inferred directly from the data provided (see above). This is now better explained in the main text (lines 518-531).

A key result of the paper concerns the role of motility in the presence of aggregates. As currently presented, the authors suggest that the rheological properties of the digesta—specifically its yield stress / gel-like behavior—facilitate the breakdown of aggregates and their displacement from the epithelium to the center of the lumen. In the abstract, they state: "In samples from mice and humans, we show that upper large-intestinal content behaves as a non-Newtonian fluid that changes its viscoelastic properties under the force of gut contractions. This phenomenon is sufficient to explain micro-scale bacterial clustering in the murine cecum, resulting from growth within the gel-like structure of cecum content, and periodic disruption due to peristalsis-driven shear-thinning and clearance." This sentence implies that the physical properties of the digesta, in the absence of flow, are sufficient to promote bacterial growth in clusters—similar to what is observed in agarose gels (Ref. 50). However, I am doubtful about the validity of this comparison. Agarose is a crosslinked polysaccharide network that traps water, whereas digesta is a suspension of solid food particles in a liquid phase. It is therefore questionable whether digesta can be reasonably described as a "gel-like" matrix in the same sense as agarose. In digesta, bacteria may experience a more water-like environment and could potentially adhere to food particles or interfaces, which may play a more significant role in clustering. These considerations remain speculative and, in my opinion, should be clearly framed as such in the discussion.

We agree that agarose gels and digesta are unlikely to share the same chemical properties, especially when it comes to the types and abundance of crosslinks. While our comparison remains phenomenological and currently lacks molecular understanding, we would still argue that the rheological properties of digesta speak for a qualitatively similar behavior, and could allow spatial confinement of growing bacteria in conditions where digesta properties are predominantly solid-like. In response to the reviewer's concerns, we have adapted the language we use in several places (lines 92-94 and 508-511).

Furthermore, in classical Newtonian fluid flow driven by circular or longitudinal gut contractions, both velocity and shear stress are typically maximal near the wall. If the digesta behaves as a yield stress fluid, this effect may be attenuated, as the flow would occur primarily in regions where the stress exceeds the yield threshold. This rheological behavior might, in fact, protect bacteria located near the wall from being displaced.

According to our rheolgical analysis, the digesta behaves as a yield-stress fluid, and because the shear stress is maximal near the wall, this is where the yield stress is most likely to be exceeded. We argue that this can contribute to more flow close to the walls, even though we do not claim that this is the only reason for increased flow in this area.

The authors' use of the Stokes' second problem to argue that oscillating contractions could overcome the yield stress and disrupt aggregates is potentially relevant, but remains a theoretical suggestion. In my view, Figure 4C convincingly supports the role of cecal motility in modulating bacterial clustering, but the role of digesta rheology remains an open question that would benefit from further experimental support.

We fully agree that the use of Stokes' second problem in this case is only a theoretical way of testing whether our hypothesis (fluid like gut content due to contractions) makes sense given the forces in the system: does the order of magnitude of length scales where the yield stress is exceeded make sense for the system we analyze? We clarify that this is only an order-of-magnitude estimation for the effect of contractile activity on gut content, given its rheological properties (lines 369-373).

We have now added experimental data on the effect of loperamide treatment, which we expected to decrease the strength of mixing in the cecum. Short-term (1.5h) treatment, however, showed no significant effect (Figure 4C), while the effect over a longer time period (6h) unexpectedly led to less bacterial clustering (Figure S8, lines 388-400 and 532-552). As we now discuss in the main text, this might have various reasons: we expect clustering to depend on bacterial growth, and 90min might not be long enough to see an effect; and the effect after 6h might be due to effects of loperamide on host physiology other than altered gut contractions, such as changes in feeding behavior (Chumakova et al, 2011) or altered flow behavior in the cecum due to obstructed outflow into the colon.

Additionally, the authors state that: "Shear-thinning can also explain the surprising observation that fed beads enter the tip of the mouse cecum by flow along the epithelial cell layer before being mixed into the cecum content." This observation, however, is not unique to shear-thinning fluids. In flow within the gut, fluid motion is driven by boundary movement, leading to maximum shear and velocity near the wall, regardless of whether the fluid is Newtonian or non-Newtonian. This well-known feature of gut biomechanics has been previously described (e.g., see Figures 3, 8, and 9 in doi:10.1098/rsif.2013.0027).

We thank the reviewer for pointing this out. While we would still argue that shear thinning can contribute to this behavior, it clearly is not the only explanation for the phenomenon, and we now acknowledge this explicitly in the text (lines 481-483, abstract). We also cite the publication the reviewer suggests, which shows that boundary movement happens whether the content is a Newtonian or non-Newtonian fluid.

Line 125:
Beads of different colors were administered at different time points. Figure 1D presents the relative abundance of beads after gavage, but the specific color coding is not described in the legend or figure. First, the histogram is somewhat difficult to interpret, as the evolution of each data ('tip center', etc.) is not clearly visible as in a standard plot. Second, additional details about bead colors and their corresponding time points would help clarify the experimental design. Have the authors attempted to plot the quantity of beads (by color/time) in each of the four anatomical segments? Such a representation could more directly support their conclusion that beads migrate along the epithelium before reaching the center of the lumen. Additionally, are these differences statistically significant?

We thank the reviewer for pointing out that the data is not easy to interpret. As both, the experimental design and the data analysis of this specific experiment are quite involved, we have now added a better description in the text (lines 154-157, legend for Figure1D,E). We have also added a figure showing the experimental setup (new Figure 1D), and have added a supplementary figure showing a different representation of the data (new Figure S3). The trends we find are not statistically significant; as the variation between mice is large, and bead distribution likely depends on many factors that are hard to control, we decided to refrain from adding many more animals to this experimental cohort, and simply show the data as it is.

Line 170:
How does the size of bacterial clusters compare to the size of a single bacterium? Is it possible to estimate the average number of bacteria per cluster? From the H(r) plots, could the authors extract or approximate a size distribution of aggregates? Given that many clusters appear to be composed of only 2-3 bacteria, this could inform whether the clusters arise from bacterial growth over time, and how this correlates with known bacterial replication rates. Such a quantitative link would strengthen the proposed growth-based mechanism of aggregation.

This question goes in a similar direction as the one above concerning the quantitative interpretation of Ripley's H function. We have added a section to the main text detailing how the radius in Ripley's H relates to real cluster sizes (lines 518-531). We agree that the small cluster sizes fit the growth-based mechanism for cluster generation, but because bacterial growth in gut lumen and contractile activity are both variables that we do not have good data for, this does not allow strong conclusions. We now discuss this in the main text (528-531).

Line 278:
The conclusion that clusters represent clonal microcolonies is supported by the data. However, the reference to "transiently unmixed cecum content" does not appear to be directly supported by the results presented. Could the authors clarify whether there is experimental evidence supporting this statement?

Based on the evidence we collected in this study we hypothesize that clusters form due to bacterial growth during the time between gut contractions, and that their small size is due to their dispersal when contractions happen. The statement that gut content is, at least locally, "transiently unmixed", reflects that hypothesis: contractions are not continuously mixing the cecum, but there are (transient) periods of time during which cecum content is unmixed. We have now changed the statment to reflect this better in the main text (lines 308-310).

Figure 4:
The description of Figure 4 in the main text does not correspondto what is actually shown in the sub-panels. The figure presents an amplitude sweep, in which both G′ and G″ plateau at low strain amplitudes, supporting the gel-like behavior of the digesta. However, the reported yield stress value should be better substantiated. Ideally, it should be supported by creep tests (i.e., the time evolution of shear strain under a constant applied shear stress), which are more

appropriate for identifying the onset of flow and determining the yield stress. I assume such tests may have been performed—if so, could the authors include them in the supplementary material to support the reported yield stress value?

We thank the reviewer for pointing this out. We have now fixed the references to Figure 4. In addition, we have made an effort to substantiate our estimation of yield stress. First, we have re-analyzed the existing oscillatory rheology data, and used the intersection point of power-law fits of shear strains vs shear stress data below and above the yielding point (new FigureS6A,B). Second, we have added new data on creep measurements that complement the yield stress estimations from our oscillatory measurements (new FigureS6C). The yield stress we obtain with creep measurements is slightly lower than what we have estimated using oscillatory measurments; this is not unexpected, as creep measuremnts are known to produce lower values for yield stress than oscillatory measurements (see e.g. Dinkreve et al, 2016). We now show the data we use to estimate yield stress in a new figure (Figure S6), and discuss this in the text (lines 338-344).

Minor comments
* Lines 51-53: The sentence is unclear and should be rephrased for clarity.

Done.

* Lines 53-54: It would be helpful to indicate the typical values or characteristic ranges of short-range interactions between bacteria.

Done.

* Line 78: Seminal work by R.G. Lentle on digesta rheology should be cited alongside references 31 and 32. Suggested references include: Lentle, R. G., & Janssen, P. W. M. (2008). Physical characteristics of digesta and their influence on flow and mixing in the mammalian intestine: a review. Journal of Comparative Physiology B, 178(6), 673-690.

We thank the reviewer for pointing out this important review, which we have now included in our citation list.

* Line 264: The sentence is unclear and needs rephrasing for clarity.

We have rephrased the sentence to increase clarity (lines 295-297).

* Line 395: The sentence appears contradictory: "In the cecum, dense mucus fills only the bottom of the intestinal crypts." Please clarify whether the mucus is localized or more diffusely distributed.

We have changed this statement to make it clear that there is no structured mucus layer covering the epithelium in the cecum, but that, due to the fact that mucus is secreted by goblet cells in the epithelium, local concentrations might still not be homogeneous. (line 479-481).

* Line 412: Could the authors clarify what is meant by "pre-existing structure"? Are they referring to structures in the physical environment (e.g., mucus, food particles) or to pre-formed bacterial colonies?

We thank the reviewer for pointing out that this was not clearly phrased, and we have changed the sentence to make the point more clearly (line 496-499).

Reviewer #3:

In this manuscript, the authors examine the spatial structure of the mouse cecum using several complimentary approaches in 3 member, 12 member and SPF conventional communities as well as oral bead delivery. They find that cecal tip as compared to the base harbor different bacteria; these differences are more pronounced than the center to epithelial axis, which I found a bit surprising. The authors next observe that the bacteria are clustered and non-random in their distribution and go on to suggest that antibody binding and bacterial chemotaxis are not responsible for this. Finally, the authors demonstrate that smooth muscle contraction/motility can influence the distribution of clusters in a non-random fashion by augmenting function with ricinoleic acid to show decreased clustering. Overall, the manuscript contains high quality data and interesting findings. The manuscript would be appropriate for this journal with some minor revisions.

We thank the reviewer for their encouraging remarks!

Strengths
-use of appropriate model systems and technology to clearly assess their questions
-A logical line of questioning with insights into physical properties influencing cecal microbiome spatial dynamics
-inclusion of human ileal effluent samples

Weaknesses
-minor weakness is focus on model system that lacks microbial diversity and may not fully reflect the biology of more complex communities.

Major Comments:
None

Minor Comments:
-As an additional experimental group to assess the role of motility in addition to increased smooth muscle function with ricinoleic acid can you administer an agent that would decrease motility (loperamide) to see if it would augment clustering?

We thank the reviewer for this suggestion, and we have now added data showing the effect of loperamide treatment on clustering of bacterial cells in cecum content (Figure 4C, Figure S8). Loperamide treatment did not show a significant effect on clustering in the short term (1.5h after treatment), which might be due to the fact that clustering depends on bacterial growth, and 1.5h might be too short to see the effect. In an additional experiment, in which we assessed the effect of loperamide 6h after treatment, the effect was striking, but unexpected: treatment decreased clustering significantly. We would argue that this could be due to a variety of factors, such as changes in feeding behavior or backflow in the cecum due to decreased flow through the colon. Supporting the idea of backflow, we see a stronger decrease in clustering in the cecum base (where inflow and outflow into the cecum happen) than in the cecum tip. We now discuss this in the text (lines 532-552).

-It would be interesting to probe their model system with addition alterations- increase volume and/or water content with osmotic agents (polyethylene glycol or psyllium husk), or alter nutrient availability with removal of fiber content or additional of purified fiber.

We fully agree that these experiments would add to our understanding of the effects. For the scope of this article, we decided to not investigate agents that act oscmotically, as the results might be hard to interpret due to the complex interactions of intestinal pressure and gut contractions.

-Fig 3C- the mutants appear to have some fitness cost (lower cell numbers). Does this result in lower total numbers or equal total numbers with more B theta and E rectale?

The experiments shown in Figure 3C and D were performed in ex-germ-free mice monocolonized with the respective E. coli strains, not in the 3MM background, so the E. coli population shown in Figure 3C is the full bacterial load in those mice. We have adapted the text to make this clearer (line 276).

-Can you include a representative image from the tip as well (either in main or supplemental)?

We have added a representative image from the tip (new FigureS1A).

-The observation that maternal transmission vs post-natal microbial acquisition has dramatic impact on IgA response is very interesting!

We agree, this will be an exciting topic for the future.

-Use of Z1331::cheY lacking chemotaxis and Z1331::flhD lacking flagella was nice

Thank you!

-Line 229 should read: we evaluated the extent of bacterial clustering in the cecum in these three cases.

We have fixed the typo and thank the reviewer for spotting it.

-I have minor concerns that in mice with a simple gnotobiotic community the enlarged cecum with altered mucus structure may limit the ability to extrapolate these data to SPF mice. This is discussed well in line 400-408 of discussion.

We thank the reviewer for this and their other remarks!
